# Patient-reported outcomes of glucagon-like peptide-1 agonists on hidradenitis suppurativa severity

**DOI:** 10.1016/j.jdin.2025.05.004

**Published:** 2025-05-29

**Authors:** Radhika Gupta, Laura Cesar, Robert Micheletti, Victoria Fang

**Affiliations:** Department of Dermatology, Perelman School of Medicine, University of Pennsylvania, Philadelphia, Pennsylvania

**Keywords:** drug response, dulaglutide, GLP-1, hidradenitis, liraglutide, medical dermatology, obesity, semaglutide, tirzepatide, weight loss

*To the Editor:* Hidradenitis suppurativa (HS) is a chronic inflammatory skin condition characterized by painful, suppurating lesions of the body folds.^1^ While its pathogenesis remains unclear, HS likely arises from immune activation around terminal hair follicles, leading to inflammation, pus formation, tissue destruction, and scarring. The association between HS and obesity has been well established, and there is data to suggest that weight loss may alleviate HS severity.^2^ Glucagon-like peptide-1 receptor agonists (GLP-1-RAs), US Food and Drug Administration-approved for diabetes and weight loss, have recently shown promise in small case studies reporting improved glycemic control and decreased HS severity over short durations.^3^, ^4^, ^5^

This cross-sectional survey study evaluates the impact of GLP-1-RAs on HS severity and quality of life using patient-reported outcomes. Adults with HS on active GLP-1-RA treatment, seen at the Department of Dermatology at the University of Pennsylvania between January 2019 and August 2024, were identified using Epic’s Slicer Dicer tool. Of 128 patients sent an electronic communication, 39 viewed the survey, and 28 completed it; 6 did not ultimately meet inclusion criteria and were excluded from further analysis.

Among the 22 participants, 90.9% were female, with an average age of 45 years (range: 29-64) (Table I). Most were Black (54.5%), non-Hispanic (90.9%), and classified as overweight or having class 1-3 obesity (89.5%). The average age at HS diagnosis was 33 years (range: 15-60). Active disease was reported by 86.4%, with 77.3% experiencing outbreaks in the past 6 months and 22.7% reporting prior hospitalizations. Menstrual flares were noted by 65.0% of female participants. The most common HS flare sites were the groin, axillae, and thighs.

**Table I:** Demographics and baseline hidradenitis suppurativa-related survey data

Age	*n* = 22	
Average	45	
Standard deviation	10	
Min	29	
Max	64	
Sex	***n* = 22**	
Female	20	90.9%
Male	2	9.1%
Ethnicity	***n* = 22**	
Not Hispanic or Latino	20	90.9%
Hispanic or Latino	2	9.1%
Race	***n* = 22**	
White	8	36.4%
Black or African American	12	54.5%
Asian	1	4.5%
Native Hawaiian or Other Pacific Islander	1	4.5%
BMI classification	***n* = 19**	
Healthy weight	2	10.5%
Overweight	2	10.5%
Class 1 obesity	1	5.3%
Class 2 obesity	5	26.3%
Class 3 obesity	9	47.4%
Age of HS diagnosis	***n* = 19**	
Average	33	
Standard deviation	14	
Min	15	
Max	60	
HS outbreak in prior 6 mo	***n* = 22**	
Yes	17	77.3%
No	5	22.7%
Hospitalization in past for HS	***n* = 22**	
Yes	5	22.7%
No	17	77.3%
Body part(s) ever affected by HS	***n* = 22**	
Head and neck	5	22.7%
Left arm pit	14	63.6%
Right arm pit	15	68.2%
Chest	10	45.5%
Abdomen	7	31.8%
Groin	19	86.4%
Back	2	9.1%
Buttocks	13	59.1%
Left thigh	14	63.6%
Right thigh	15	68.2%
Other	3	13.6%
Flare around menstrual cycle among females	***n* = 20**	
Yes	13	65.0%
No	7	35.0%
Current status of HS	***n* = 22**	
Clear - 0	3	13.6%
Almost clear - 1	4	18.2%
Mild disease - 2	6	27.3%
Moderate disease - 3	4	18.2%
Severe disease - 4	4	22.7%
Biggest HS concern	***n* = 20**	
Skin symptoms - itch, pain, stinging	11	55.0%
Skin appearance	6	30.0%
Worry about what else might be happening	1	5.0%
Other symptoms - fatigue, fevers, infections, feeling poorly in general	2	10.0%

The bolded values in the table represent the number of participants who provided responses to that particular question in the survey.

*BMI*, Body mass index; *HS*, hidradenitis suppurativa.

Participants were prescribed semaglutide (40.9%), tirzepatide (36.4%), dulaglutide (18.2%), or liraglutide (4.5%), primarily for weight loss or diabetes, not HS (Table II). The average treatment duration was 17 months (range: 2-108). No association between duration of treatment and change in HS-specific health was demonstrated. Most participants (77.3%) reported weight loss, averaging 31 pounds. Notably, 68.2% observed improved HS-specific health, with the remainder (31.8%) reporting no change. Patients reported a reduction in flares (61.9%), new lesions (66.7%), pain (52.4%), drainage (61.9%), itch (47.6%), and odor (42.9%). A total of 59.1% reported less impact of HS on daily activities. Common side effects included gastrointestinal symptoms, allergic reactions, headaches, menstrual spotting, and appetite suppression. Overall, 59.1% indicated they would recommend GLP-1-RAs to other HS patients.

**Table II:** Glucagon-like peptide-1 receptor agonist-related survey data

Prescribed agent (*n* = 22)					
Semaglutide (brand names: Ozempic, Rybelsus, Wegovy)	9	40.9%			
Dulaglutide (brand name: Trulicity)	4	18.2%			
Liraglutide (brand names: Victoza, Saxenda)	1	4.5%			
Tirzepatide (brand names: Zepbound, Mounjaro)	8	36.4%			
Indication(s) for GLP-1-RA prescription (***n = 22***)					
HS	0	0.0%			
Weight loss	8	36.4%			
Diabetes	8	36.4%			
Diabetes + weight loss	6	27.3%			
Time on agent (in mo) (***n = 16***)					
Unspecified	3				
<1 month	3				
Average	17				
Min	2				
Max	108				
Overall improvement in HS-specific health on GLP-1-RA (***n = 22***)					
No change	7	31.8%			
Minimally improved	6	27.3%			
Much improved	5	22.7%			
Very much improved	4	18.2%			
Change in HS-related symptoms since starting GLP-1-RA (***n = 21***)					
	*1 - Much worse*	*2 - Worse*	*3 - Unchanged*	*4 - Improved*	*5 - Much improved*
Pain	0	0	10	7	4
Itch	0	1	10	6	4
Drainage	0	0	8	10	3
Odor	0	0	12	6	3
	*1- Many more lesions*	*2 - More lesions*	*3 - Unchanged*	*4 - Fewer lesions*	*5 - Much fewer lesions*
New lesions	0	0	7	7	7
	*1 - Many more flares*	*2 - More flares*	*3 - Unchanged*	*4 - Fewer flares*	*5 - Much fewer flares*
Flares	0	0	8	6	7
Effects of HS on activities since starting GLP-1-RA (***n = 22***)					
Unchanged	9	40.9%			
Less bothered	8	36.4%			
Much less bothered	5	22.7%			
Weight loss on GLP-1-RA (***n = 22***)					
Yes	17	77.3%			
No	4	18.2%			
Unsure	1	4.5%			
Weight loss on GLP-1-RA (in lbs) (***n = 17***)					
Average	31				
Min	3				
Max	75				
Recommendation of GLP-1-RA to other HS patients (***n = 22***)					
Yes	13	59.1%			
No	2	9.1%			
Unsure	7	31.8%			

The italicized values for the question “Change in HS-related symptoms since starting GLP-1-RA” represent the Likert scale values. For example, for the change in “new lesions,” participants were asked to respond to the question on a Likert scale of 1 (many more lesions) to 5 (much fewer lesions).

*GLP-1-RA*, Glucagon-like peptide-1 receptor agonist; *HS*, hidradenitis suppurativa.

These data suggest GLP-1-RAs may play an important adjunctive role in the treatment of HS, particularly given the high prevalence of obesity and diabetes in the HS population. Our study is limited by the small sample size, lack of clinician assessment, high nonresponse rate, and potential recall bias; however, a strength of this study is the focus on patient-reported experience. Future randomized controlled studies with robust patient and dermatologist-reported endpoints are needed to confirm these findings and establish clinical guidelines for use of GLP-1-RAs in hidradenitis.

## Conflicts of interest

Author Gupta is a consultant for Cabaletta Bio. Dr Micheletti is a consultant for Vertex and reports research funding from Amgen, Boehringer Ingelheim, Cabaletta Bio, and InflaRx. Dr Fang is a consultant for Sonoma Biotherapeutics. Author Cesar no has conflicts of interest to declare.
